# The New Nurses' Prospects

**Published:** 1919-06-14

**Authors:** 


### THE MATRONS' AND SISTERS' DEPARTMENT.

### THE NEW NURSES' PROSPECTS.

The Report of the Salaries Committee of the College of Nursing, which has set an immense deal of discussion buzzing during the last week, opens up an interesting vista of future nurses' pay and prospects. For, though there are many estimable and experienced persons at work, declaring that the conclusions arrived at and the recommendations made in respect of salaries are futile and impossible, yet the world does move and the impossibilities of to-day become the accepted basis of operations to-morrow. We shall not go far wrong if we assume that the suggestions made in the report are likely to be in general force within the next ten years.

Just before this report was issued the Liverpool hospitals agreed upon a scheme of affiliation which is likely to have far-reaching consequences. The general hospitals with a four-year course agreed to reduce this course to three years for a certain number of pupils who had already received two years' preliminary training in one of the special hospitals affiliated to them. Within five years, according to this scheme, nurses can obtain a full certificate of training from a first-rate general hospital, together with such special certificates as will certainly put them well forward in their career, either in private nursing or administrative posts.

The Salaries Report suggests that the wage of a probationer entering a special hospital at the age of eighteen should be £12 a year, together with uniform allowance, laundry, and full board and lodging. She serves two, or possibly, in some cases, three years at £12 to £15, with emoluments which make the salary in money value not less than £100 a year. She then passes on to a general hospital, where, at the age of twenty or twenty-one, she obtains a salary of £20, rising to £22 in her second year, and £30 in her third. As a fully trained nurse she now receives £50, with emoluments equal to £100 a year. How do her prospects compare with those of her brothers and sisters who enter business life or become clerks?

There are very few openings for either men or women in which the learner is self-sufficing from the first moment of leaving school while able to command the standard of living maintained in nurses' homes. The ordinary clerk often commences at £60 and is lucky if by the time his sister has become a fully-trained nurse at twenty-three he is earning as much as £150. He receives no emoluments, and has often an expensive journey to his office day by day. He has hard work to make both ends meet, and if he lives at home usually contributes less than his average share of the household expenses. Promotion is very slow. He may venture to marry on £200 or £250 a year, but that will not be his before he is thirty. The girl clerk's prospects are even less rosy. If she has no home she lives in rooms, or at a cheap hostel, under conditions which sap her energies and render her life a remorseless struggle to make both ends meet. If she is just one of the rank and file, like thousands of other girls, she will get no chance of promotion, and her maximum will stop at, say, £130, for work of an entirely monotonous character which satisfies none of her aspirations. And, in spite of all thwartings, aspirations persist, and when unsatisfied turn to bitterness and disgust.

The nurse, on the other hand, who at twenty-three is earning a salary of £50, with every want supplied, has a good future before her even if she has no conspicuous gifts. Her training has enabled her to turn every ounce of good stuff in her nature to good account. She is mistress of herself. She may stay in institutions and rise to a salary of £75 as ward sister; she may take up private nursing and earn from £100 to £120 upwards; or she may become a district nurse and earn from £60 to £75, in all cases free money, with board and lodging, often uniform also, provided. This for the quite ordinary, not remarkably clever, women with which the training-schools are filled. To this a secure pension may soon be added.

If we take exceptional women, it cannot be denied that there are chances of getting rich in a business career which do not come in the way of nurses, though such chances generally demand capital. But, if the recommendations of the Salaries Committee are carried out, there will be a large number of appointments thrown open to nurses of sufficient value to satisfy all reasonable expectations. The salaries recommended for matrons start at £200 for institutions with over one hundred occupied beds, and rise to £350 for those over five hundred beds. The emoluments which go with these salaries are admittedly first-rate. And, short of matronships, there are hundreds of institution posts commanding salaries between £100 and £130, besides the big prizes of successful private work and superintendents' posts in other branches of the nursing service. In short, the new scale of salaries suggested by the College of Nursing should wipe out the reproach that the nursing service is the worst-paid profession open to women. This desirable end should be speedily brought about by the nurses actively interesting themselves in the elections to the Council of the College. More vigorous activity and knowledgeable work on the part of the College Council will be welcome. The electorate possess plenty of ginger; let them use it freely and continuously until the business of the Council begins to move with a hum.

